# Case Report: Conservative Treatment of Adolescent Idiopathic Scoliosis Can Alter the Perception of Verticality. A Preliminary Study

**DOI:** 10.3389/fped.2020.609555

**Published:** 2021-01-25

**Authors:** Noelia Zagalaz-Anula, Felipe León-Morillas, Juan Alfonso Andradre-Ortega, Alfonso Javier Ibáñez-Vera, Silvana Loana de Oliveira-Sousa, Rafael Lomas-Vega

**Affiliations:** ^1^Department of Health Sciences, University of Jaen, Jaen, Spain; ^2^Health Sciences PhD Program, Catholic University of Murcia (UCAM), Guadalupe, Spain; ^3^Department of Physical Medicine and Rehabilitation, Hospital Complex of Jaen, Jaen, Spain; ^4^Department of Physiotherapy, University of Murcia, Murcia, Spain

**Keywords:** idiopathic scoliosis, postural control, adolescent idiopathic scoliosis, scoliosis physical therapy, sense of verticality, spinal diseases

## Abstract

Adolescent idiopathic scoliosis (AIS) is a lateral curvature of the spine of at least 10° Cobb's angle of unknown etiology. Some studies have found that patients with AIS have a Visual Verticality (VV) perception similar to healthy controls. This study aimed to analyze VV perception and postural balance differences in patients with AIS depending on the management, either based on observation or conservative treatment. Eighteen patients with AIS were included in this study. Nine patients were managed based on observation. The other nine underwent conservative treatment, such as bracing or exercise. Subjective Visual Vertical (SVV) and posturographic parameters were measured and analyzed. In the SVV test, patients who underwent treatment showed poor constant error in absolute values and mean absolute error, with statistically significant differences (*p* < 0.05). Only the Romberg Quotient for sway area was within the limits of statistical significance for posturographic parameters, with a lower value for patients under observation. This study found worse perception of verticality in patients receiving some type of conservative treatment than patients receiving only observation; whereas posturography showed similar values in both observation and treatment groups. Our results can be interpreted as the effect of treatment on the previous verticality perception adapted to the curvature.

## Introduction

Adolescent idiopathic scoliosis (AIS) is a lateral curvature of the spine of at least 10°, measured with Cobb angle, of unknown etiology occurring in children and adolescents aged 10–18 years (1). AIS is the most common form of scoliosis and is distinguished from other types of scoliosis by the absence of underlying congenital or neuromuscular abnormalities (2). In the United States, the estimated prevalence of AIS with a Cobb angle of at least 10° among children and adolescents aged 10–16 years is 1–3% (3). The development of spinal deformity may impact a patients' level of reported quality of life (QOL) (4).

Various theories have hypothesized the causes of AIS, including biomechanical, neuromuscular, genetic, and environmental origins; yet our understanding of scoliosis etiology is still limited. There is moderate-quality evidence for decreased postural stability in AIS measured with the following Center of Pressure (CoP) parameters: sway area, and medio-lateral and antero-posterior range with a positional shift posteriorly in the sagittal plane (5). Another study (6) found that in most patients with AIS, Subjective Postural Vertical (SPV) was shifted to the right, with no alteration of the Subjective Visual Vertical (SVV). Catanzariti et al. (7) proposed a global pathophysiological concept, which involves an issue with orthostatic postural control, with disturbance in the multisensory integration of vestibular, visual, and somesthetic inputs. Thus, AIS could be the consequence of a reorientation of the longitudinal body axis in accordance with an erroneous central representation of verticality (7). Another author proposed that AIS might be the consequence of a reoriented longitudinal body axis aligned with an erroneous vertical reference (6).

Nonetheless, the evidence investigating the effect of different therapies on the most used tests of verticality, such as SVV and postural control in patients with AIS, are scarce. For this reason, the primary aim of this study was to describe these variables in patients with AIS who underwent different types of conservative management, such as observation only, exercise, or brace orthosis. Additionally, we compared the different types of management approaches and their influence on posture perception.

## Method

### Participants

This case series selected patients who were treated in the Tertiary Hospital of Jaen (Andalusia, Spain) or private clinics specializing in idiopathic scoliosis in Jaen City and province. Selection criteria were patients diagnosed with AIS, aged 10–18 years old and with a Cobb angle >10° who received conservative treatment, or observation only. Patients with neuromuscular problems and surgical treatment were excluded. The research protocol was approved by the Ethics committee of the Jaen Hospital (Code VEFRE-30/05/19), and the study was designed and conducted in accordance with the Ethics Code of the World Medical Association for studies including human participants (Declaration of Helsinki). All participants were fully informed regarding the details of the study and were required to provide written informed consent demonstrating their voluntary agreement to participate in the study along with the agreement and consent of their legal guardians.

### Therapeutic Management

Patients underwent different types of management in function of their curve status. Patients with a slight curve generally underwent exercise treatment by GPR (Global Postural Reeducation). This method consists of carrying out self-stretching exercises, keeping the anteroposterior curves of the spine within physiological limits, and correcting the lateral curves using a type of breathing called “paradoxical” that involves the active descent of the diaphragm during exhalation and contraction of the oblique abdominal muscles to remove the anterior and posterior rib humps. This method is based on an initial physical evaluation, whose reliability has been evaluated (8). Patients with moderate curvatures were treated with a Cheneau Brace. This type of brace orthosis has been shown to be effective in treating scoliosis in the mid to long term (9) and may produce relevant changes in CoP sway (10).

### Measurements

Patients were diagnosed by a physician specialized in Physical Medicine and Rehabilitation per the criteria of AIS as a tridimensional curvature with at least 10° of Cobb angle measured with anteroposterior X-ray.

**Figure 1:**
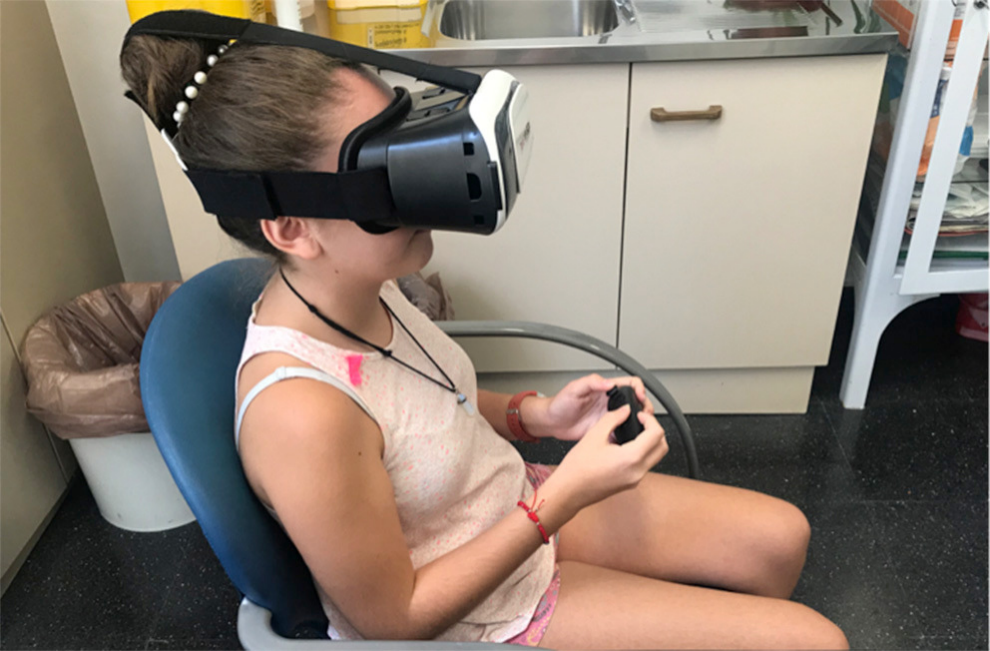
SVV measurement with a device based on virtual reality.

The SVV test is the main test used to assess verticality in clinical research (11). This tool assesses the body position of the subject with respect to gravity. For the SVV measurement, a device based on virtual reality—developed at the University of Jaen and previously validated—was used (12). The subject is placed in a seated position (13) wearing virtual reality glasses; in them, the participant is asked to position a luminous bar that is surrounded by darkness at 0° with no visual reference, starting the bar at 60, 45, and 30° to the right and left alternately (12). Six measurements were taken for each subject in the sitting position (14) (Figure 1). The constant error was calculated as the average value of the error made in each attempt, with a negative sign if the error was to the left and positive if the error occurred due to a deviation to the right. The mean absolute error was calculated as the average of the deviations from the true unsigned vertical. A third measurement was obtained from eliminating the ± sign of the constant error (constant error in Absolute value).

To evaluate postural control, posturographic parameters were obtained with a resistive multisensor platform (Sensor Medica, Rome, Italy) with an active surface of 400 × 400 mm and an acquisition frequency of 40 Hz. Calculations of CoP movements were performed using the Free-Step Standard 3.0 software (Italy). Posturographic parameters related to the global dispersion of the Body Pressure Center (CoP) were measured, including the Oscillation Area (Sway Area) and its Average Speed (V), mediolateral (Xmean) and anteroposterior (Ymean) mean position parameters, and the length of the CoP path. These parameters were obtained in two tests with Open Eyes (EO) and Closed Eyes (EC), per the previously established procedure (15). From these parameters, the Romberg quotient for Sway Area (Sway Area OC/Sway Area OA) and Speed was obtained. Participants stood barefoot, as still as possible, with their arms at their sides, feet at a 30° angle, and heels 2 cm apart. In the tests performed with EO, the participants looked at a fixed point located on the wall at eye level.

### Data Analysis

Data were described by means and standard deviation for continuous variables and by frequencies and percentages for categorical variables. The Kolmogorov-Smirnov test was used to evaluate the normality of the data, and the Levene test was used for homoscedasticity. Differences between groups of subjects followed or treated were analyzed by a multivariate linear general model with the group (observation only or treated) as a factor and curve degrees as a covariable. Dependent variables were SVV test outcomes and posturographic parameters. The coefficient of determination R^2^ was used as an effect size measure. According to Cohen (16), R^2^ can be classified as insignificant when it is <0.02; small if it is between 0.02 and 0.15; medium if it is between 0.15 and 0.35; large if >0.35. Data handling and analysis were conducted using the statistical package for social sciences version 21 (SPSS Inc., Chicago, IL, USA). The confidence level was set at 95% (*p* < 0.05).

## Results

Eighteen patients were recruited and evaluated after applying the selection criteria. Nine patients underwent conservative treatment (four orthotic and five exercises), and nine were under observation only. Sociodemographic characteristics are shown in Table 1.

**Table 1:** Sociodemographic characteristics of the study sample.

		**Sample (*****n*** **= 18)**				**Observation (*****n*** **= 9)**				**Conservative treatment (*****n*** **= 9)**			
**Variables**		**Mean**	***SD***	***F***	**%**	**Mean**	***SD***	***F***	**%**	**Mean**	***SD***	***F***	**%**
Age		13.39	1.42			13.33	0.71			13.44	1.94		
Height		159.33	9.43			162.33	10.03			156.33	8.261		
Weight		53.78	11.91			54.72	10.85			52.83	13.47		
Body mass index		21.21	4.68			20.77	3.71			21.66	5,68		
Main curve degrees		17.44	7.70			14.00	3.76			20.89	9.23		
Gender	Male			4	22.2			4	44.44			0	0
	Female			14	77.8			5	55.56			9	100
Type	King II			7	38.9			2	22.22			5	55.56
	King IV			6	33.3			4	44.44			2	22.22
	King I			5	27.8			3	33.33			2	22.22
Side of the curve	Right thoracic			12	66.7			5	55.56			7	77.78
	Left thoracic			6	33.3			4	44.44			2	22.22
Treatment	Observation			9	50.0			9	100			0	0
	GPR			5	27.8			0	0			5	55.56
	Orthosis			4	22.2			0	0			4	44.44

*F, Frequency; GPR, Global Postural Reeducation; Observation, under observation only; SD, standard deviation*.

In terms of SVV measures (Table 2), differences in constant error (with ± sign) were not statistically significant; however, in descriptive analysis, patients who underwent conservative treatment showed good alignment of SVV for the true vertical, but patients who were under observation only showed a deviation of the SVV to the left. Conversely, patients who underwent treatment showed poor constant error in absolute values and in mean absolute error, with statistically significant differences (*p* < 0.05).

**Table 2:** Differences in SVV measures.

**Visual verticality**	**Observation**		**Conservative Treatment**		***p*-Value**	**Effect**
	**Mean**	***SD***	**Mean**	***SD***		
SVV_Constant_Error	−1.87	2.25	0.08	8.65	0.319	0.066
SVV_Constant_Error_Abs	2.49	1.41	6.59	5.10	0.014^*^	0.341
SVV_Mean_Absolute_Error	4.50	6.31	10.88	14.05	0.045^*^	0.241

*Observation, under observation only; SD, standard deviation; SVV, Subjective Visual Vertical*.

* *p < 0.05*.

No significant results were obtained for posturographic parameters (Table 3). Only the Romberg Quotient for Sway Area neared the limits of statistical significance, with a lower value for patients under observation only, which could be interpreted as less influence of vision in postural balance.

**Table 3:** Differences in posturographic parameters.

**Parameters**	**Observation**		**Conservative Treatment**		***p*-Value**	**Effect Size**
	**Mean**	***SD***	**Mean**	***SD***		
Sway area EO (mm^2^)	64.30	41.99	31.68	22.92	0.085	0.184
Length sway EO (mm)	531.56	107.69	512.64	164.05	0.793	0.005
Velocity EO (mm/s)	8.86	1.81	8.68	2.62	0.705	0.010
CoP mediolateral EO (mm)	−2.74	8.25	−2.77	3.47	0.479	0.034
CoP anteroposterior EO (mm)	−13.56	23.06	−30.83	14.84	0.117	0.156
Sway area EC (mm^2^)	70.06	70.06	39.69	20.97	0.356	0.057
Length sway EC (mm)	530.93	156.61	570.12	146.59	0.424	0.043
Velocity EC (mm/s)	8.83	2.61	9.64	2.29	0.347	0.059
CoP mediolateral EC (mm)	−3.23	7.46	−0.02	3.40	0.189	0.112
CoP anteroposterior EC (mm)	−22.28	17.74	−26.11	11.92	0.520	0.028
Romberg quotient sway area	1.21	0.82	1.70	0.71	0.076	0.195
Romberg quotient velocity	0.99	0.20	1.13	0.24	0.332	0.063

*Observation, under observation only; CoP, Center of Pressure; EC, Eyes Closed; EO, Eyes Open; SD, standard deviation*.

## Discussion

This study's objective was to describe the sense of verticality measured with SVV test and postural control in patients with AIS, comparing these variables in different types of conservative management: under observation only, therapeutic exercise only, or orthotic brace only. The overall results showed no differences in postural control measured by posturographic parameters. However, there appeared to be differences in verticality perception between different conservative treatments. Concretely, it appears that the conservative treatment applied to the scoliotic curve provoked poor results in verticality perception compared to solely observation. This could be interpreted as the disruptive effect that the treatment produces on the perception of verticality of patients with AIS, who are previously adapted to perceive good verticality while living with the scoliotic curve. At the same time, based on these observations, the results of this study support that conservative treatments for the scoliotic curves influence the posture perception of patients with AIS. These conservative treatments include well-designed and adapted braces providing 50% correction, applied in progressive curves between 20 and 45° (17), and Schroth exercises in those patients with AIS with a Cobb's angle between 10 and 30°. However, evidence does not support this latter as more effective than traditional exercises (18). There are other methods based on respiratory exercise, such as Global Postural Reeducation (GPR), that has proven to be effective in treating spinal pain (19), but its efficacy in treating scoliosis is at an early stage (20), and the evidence is still scant.

Available evidence is scarce concerning the treatment of verticality perception and postural control in AIS. As the results of this study showed worse perception of verticality in patients receiving some type of conservative treatment, our hypothesis is that with the progression of the scoliotic curve, a compensatory mechanism for the altered SVV occurs, appearing as a mismatch in the SVV while the scoliotic curve is being corrected. In a clinical trial conducted by Yagci et al. (21), subjective vertical visual perception only improved significantly (*p* < 0.05) in patients with AIS who performed body awareness exercises; however, this did not happen in those patients with AIS who performed Core Stabilization Exercises or Traditional Exercises. In contrast, there was a significant improvement (*p* < 0.05) in subjective visual horizontal perception, postural vertical perception, total postural perception scores, total haptic perception scores, and haptic perception 45° to the right by the use of the stabilization and body awareness exercises. This indicates that body awareness exercises significantly improve SVV and that perception of verticality and postural perception play an important role in AIS. Another study found that wearing a spinal brace improved postural stability in terms of increased proprioception, equilibrium performance, and rhythmic movement ability in patients with AIS (22), during the time of wearing the orthosis. However, both studies had small samples, and their results can be considered preliminary.

Additionally, in this study, only the Romberg Quotient for Sway Area of posturographic parameters neared the limits of statistical significance with a lower value for the patients being followed-up, which could be interpreted as less influence of vision in postural balance. Overall, in our study, no differences were found in postural control according to the type of treatment the participants received. However, significant differences appeared in the variables related to SVV. This fact is very significant because it indicates that subjects with scoliosis can establish different strategies to maintain adequate control of posture while living with the curve. While untreated patients have good postural control, good perception of verticality, and scoliotic curves, treated subjects appear to enter a different state in which they obtain good postural control by distorting their perception of verticality to fit the curves.

If the data observed by this study are confirmed, it could mean that the treatment of scoliosis produces a distortion of the perception of verticality of the subjects, whose postural control system would tend to recover while maintaining the scoliotic curves, producing resistance to the improvement of the deformity. If this hypothesis is confirmed, the effect of the readjustment of the perception of verticality should be explored to progressively adapt it to the curve's improvement, thus consolidating the results of the treatment. Treatments linking the correction of the scoliotic curve and the management of postural control and SVV could be the key to obtain success in this pathology.

Some limitations of this study must be considered when interpreting the results. The sample recruited in the present study was limited, and the treatment was carried out heterogeneously (brace orthosis and GPR). Furthermore, our research was performed with adolescents from a specific geographic area, and generalization of its results may be limited to individuals with characteristics similar to those of our sample population. Moreover, the cross-sectional nature of our design does not allow causal relationships to be established between postural control. At our best knowledge, the sitting measurement of perception of verticality is not the only way for assessment of the SVV in scoliosis, so it cannot be assured that it is the best way. In this line, future studies should compare different measuring positions, such as standing or standing on foam ground. Forthcoming research should explore prospective designs with a larger and more diverse population, using objective measures to evaluate these variables.

In conclusion, the results of this study showed a worse perception of verticality in patients receiving some type of conservative treatment compared to patients only under observation. Furthermore, only the Romberg Quotient for Sway Area of posturographic parameters showed lower values for the patients under observation, which could be interpreted as a lower contribution of vision in postural balance; however, this result was not significant in our study.

## Data Availability Statement

The raw data supporting the conclusions of this article will be made available by the authors, without undue reservation.

## Ethics Statement

The studies involving human participants were reviewed and approved by Ethics committee of the Jaen Hospital (Code VEFRE-30/05/19). Written informed consent to participate in this study was provided by the participants' legal guardian/next of kin. Written informed consent was obtained from the minor(s)' legal guardian/next of kin for the publication of any potentially identifiable images or data included in this article.

## Author Contributions

NZ-A, JA-O, and SO-S: conceptualization. NZ-A, JA-O, and RL-V: methodology. RL-V: software, formal analysis, and writing—review and editing. AI-V: investigation. FL-M, AI-V, and JA-O: resources. FL-M and AI-V: data curation. NZ-A: writing—original draft preparation. SO-S: supervision. NZ-A and FL-M: project administration. All authors have read and agreed to the published version of the manuscript.

## Conflict of Interest

The authors declare that the research was conducted in the absence of any commercial or financial relationships that could be construed as a potential conflict of interest.
